# Dietary supplementation of α-linolenic acid induced conversion of n-3 LCPUFAs and reduced prostate cancer growth in a mouse model

**DOI:** 10.1186/s12944-017-0529-z

**Published:** 2017-07-11

**Authors:** Jingjing Li, Zhennan Gu, Yong Pan, Shunhe Wang, Haiqin Chen, Hao Zhang, Wei Chen, Yong Q. Chen

**Affiliations:** 1 State Key Laboratory of Food Science and Technology, Jiangnan University, Wuxi, 214122 People’s Republic of China; 2 School of Food Science and Technology, Jiangnan University, Wuxi, 214122 People’s Republic of China; 3 Beijing Innovation Centre of Food Nutrition and Human Health, Beijing Technology and Business University (BTBU), Beijing, 100048 People’s Republic of China; 4 Department of Cancer Biology, Wake Forest School of Medicine, Winston-Salem, NC 27157 USA

**Keywords:** α-linolenic acid, N-3 LCPUFAs, Conversion, Prostate cancer

## Abstract

**Background:**

α-linolenic acid (ALA) is an n-3 polyunsaturated fatty acid (PUFA) and the substrate for long-chain n-3 PUFAs. The beneficial effects of ALA on chronic diseases are still in dispute, unlike those of eicosapentaenoic acid (EPA) and docosahexaenoic acid (DHA).

**Methods:**

The primary objective of this investigation was to evaluate the efficiency of ALA uptake from a vegetable oil source and its subsequent conversion to n-3 long-chain PUFAs (LCPUFAs) in the tissues of growing mice, and to investigate its protective role in a prostate cancer animal model. We carried out the investigation in prostate-specific *Pten*-knockout mice with specified low-ALA (L-ALA, 2.5%) and high-ALA (H-ALA, 7.5%) diets. Total fatty acids in blood, liver, epididymal fat pad, prostate were detected and prostate weight were adjusted for body weight (mg/25 g).

**Results:**

We found that dietary ALA triggered significant increases in ALA, EPA, docosapentaenoic acid (DPA) and DHA levels and a significant decrease in arachidonic acid levels during the mice’s growth stage. A dose-dependent effect was observed for ALA, EPA and DPA, but not DHA. Furthermore, the average prostate weights in the L-ALA and H-ALA groups were lower than those in the control and n-6 groups, and similar to those in the EPA and n-3 groups.

**Conclusions:**

Our data suggest that dietary supplementation with ALA is an efficient means of improving n-3 LCPUFAs in vivo, and it has a biologically effective role to play in prostate cancer, similar to that of fish oils.

**Electronic supplementary material:**

The online version of this article (doi:10.1186/s12944-017-0529-z) contains supplementary material, which is available to authorized users.

## Background

In mammals, n-3 and n-6 polyunsaturated fatty acids are essential fatty acids that cannot be synthesised de novo and must be obtained through dietary sources. The n-3 long-chain polyunsaturated fatty acids (LCPUFAs), principally eicosapentaenoic acid (EPA; 20:5 n-3) and docosahexaenoic acid (DHA; 22:6 n-3) in cell membranes play an important role in determining cell and tissue function. Substantial experimental and epidemiological studies have proved that dietary interventions with n-3 LCPUFAs have preventive and/or treatment effects on inflammatory and cardiovascular diseases and some cancer types, including breast, prostate, colon, skin, and lymphoma [1–5]. As a consequence, increased consumption of EPA and DHA through fish or fish oil is highly recommended [6]. Unfortunately, fish sources are less accessible than plant sources.

α-linolenic acid (ALA; 18:3 n-3) is usually the major n-3 PUFA in the diet, and is present in some vegetable oils including flaxseed (ALA contents>50%), canola and soybean [7]. A wide range of plant products, such as nuts, seeds, vegetables, legumes, grains, and fruits, also contribute to the total ALA intake [8]. Although ALA serves as a precursor for EPA and DHA, whether it has anti-inflammatory effects and the same effectiveness as its elongated metabolites is a controversial and conflicting issue. A study that followed a cohort of 47,866 US men aged 40–75 y with no cancer history for 14 y suggested that increased dietary intake of ALA may increase the risk of advanced prostate cancer [2]. Another study comparing the effect of manipulating the n-6/n-3 ratio utilising ALA, EPA and DHA found that reducing the n-6/n-3 ratio with ALA was not as effective as EPA and DHA for mitigating obesity or the development of type II diabetes mellitus [9]. However, Oro Inca oil (rich in ALA) supplementation in Walker 256 tumour-bearing rats inoculated with Walker 256 tumour cells did cause a reduction in tumour mass and tumour cell proliferation and avoided cancer cachexia effects similar to n-3 LCPUFAs [10]. Similarly, a study that compared the effects of ALA-rich chia oil and fish oils enriched with either EPA or DHA on cardiovascular, hepatic and metabolic parameters in a diet-induced rat model of the human metabolic syndrome found that ALA did reduce inflammation. It therefore seems likely that ALA has independent effects that do not rely on its metabolism to DHA [11].

As with other dietary-derived PUFAs, a high percentage of ALA uptake from the diet results in β-oxidation of approximately 60–80%, which means its conversion rate is relatively poor [12, 13]. However, extensive dietary sources make it easy to access plenty of ALA. In our previous study, in which 0–10 wt% of ALA was added to the diet, we demonstrated that mice effectively absorbed ALA as a dietary supplement. As mice are able to absorb ALA, it is necessary to consider whether the dietary ALA absorbed can be efficiently converted into LCPUFAs and whether the stored ALA and/or the converted long-chain metabolites have a biological effect. Diet is considered one of the major modifiable environmental factors influencing prostate cancer progression [14]. In particular, dietary fat has received more attention than any other dietary component [15]. Our earlier study demonstrated that n-3 LCPUFAs inhibited prostate cancer growth and development in *Pten*-knockout mice, which develop prostate cancer spontaneously [1]. Therefore, we used prostate-specific *Pten*-knockout mice fed an ALA-enriched diet with defined PUFA levels to investigate the influence of dietary ALA on the level of n-3 LCPUFAs in vivo and its effect on tumour growth.

## Methods

### Mice

Prostate-specific *Pten*-knockout mice were generated as described previously (*n* ≥ 12 per experimental group) [1]. All animals were housed in the Animals Housing Unit of Jiangnan University at a room temperature of 23 ± 1 °C with a 12 h light/dark cycle. The mice were housed in individual ventilated cages and sterile water was provided ad libitum. F_1_ and F_2_ mice were maintained on the specified experimental diets, and only F_2_ males were used in our experiments. The F_2_ males were sacrificed at the age of the 8 weeks and a section of prostate was removed and frozen at −80 °C. Animal care and experimental protocols were conducted in compliance with the Animal Ethics Committee of Jiangnan University, China, and were performed according to the ethical guidelines of the European Community ethical guidelines (Directive 2010/63/EU).

### Diets

All of the experimental diets consisted of 13.0% fat, 46.5% carbohydrate, 24.5% protein and 11% alphacel (as the source of dietary fibre). The diet formulas are provided in Additional file 1: Table S1. The L-ALA diet and H-ALA diet contained 2.5% and 7.5% ALA by weight percent, respectively, which mainly came from flaxseed oil. The percentage of ALA in the flaxseed oil was 82.8%. All feed was kneaded into balls and stored at −20 °C before being given to the mice. The fatty acid profiles of the diets are presented in Table 1. Briefly, there was a predominance of oleic acid (53.0%) in the control diet and of LA (39.9%) in the n-6 diet. The L-ALA diet contained 17.2% ALA and 13.4% LA. The H-ALA diet was rich in ALA (43.4%), and contained 16.7% LA. The EPA diet contained 12.9% EPA and 1.6% DHA. In the n-3 diet, the ratio of EPA:DHA was 1, with the percentage reaching 7.0%.

**Table 1:** Fatty acid profile of the experimental diets^a^

	Diet^c^, %					
Fatty acid	CON	L-ALA	H-ALA	EPA	n-6	n-3
14:0	1.09 ± 0.01	1.13 ± 0.08	0.75 ± 0.03	1.06 ± 0.00	1.15 ± 0.07	1.17 ± 0.03
15:0	0.09 ± 0.00	0.09 ± 0.01	0.08 ± 0.01	0.09 ± 0.00	0.10 ± 0.10	0.09 ± 0.01
16:0	24.9 ± 0.20	24.7 ± 0.26	13.8 ± 0.45	21.2 ± 0.23	23.2 ± 0.16	25.2 ± 0.18
16:1	0.97 ± 0.02	0.51 ± 0.03	0.31 ± 0.01	0.58 ± 0.01	0.25 ± 0.02	0.61 ± 0.03
17:0	0.15 ± 0.01	0.12 ± 0.01	0.08 ± 0.00	0.13 ± 0.03	0.13 ± 0.00	0.13 ± 0.01
18:0	6.26 ± 0.08	4.96 ± 0.16	2.86 ± 0.06	5.48 ± 0.06	5.66 ± 0.02	5.35 ± 0.13
18:1	53.0 ± 0.11	36.7 ± 0.30	21.3 ± 0.02	37.2 ± 0.40	28.0 ± 0.14	39.3 ± 0.16
18:2	11.9 ± 0.07	13.4 ± 0.01	16.7 ± 0.13	15.5 ± 0.10	39.9 ± 0.08	10.6 ± 0.10
18:3	0.60 ± 0.02	17.2 ± 0.12	42.4 ± 0.61	1.16 ± 0.03	0.39 ± 0.04	0.44 ± 0.01
20:0	0.67 ± 0.01	0.93 ± 0.13	1.68 ± 0.20	0.56 ± 0.01	0.58 ± 0.01	0.51 ± 0.02
20:1	0.31 ± 0.01	0.19 ± 0.01	0.12 ± 0.01	0.47 ± 0.01	0.23 ± 0.01	0.34 ± 0.02
20:4 n-6	ND^b^	ND	ND	0.97 ± 0.03	ND	0.57 ± 0.02
20:4 n-3	ND	ND	ND	0.60 ± 0.01	ND	0.31 ± 0.01
20:5	ND	ND	ND	12.9 ± 0.41	ND	7.03 ± 0.15
22:0	ND	ND	ND	0.20 ± 0.02	0.35 ± 0.03	ND
22:5 n-6	ND	ND	ND	ND	ND	0.58 ± 0.00
22:5 n-3	ND	ND	ND	0.38 ± 0.01	ND	0.69 ± 0.01
22:6	ND	ND	ND	1.62 ± 0.11	ND	7.01 ± 0.08

^a^Values are means ± SEMs (*n* = 3)

^b^ND, not detected

^c^Data expressed as percentage of total fatty acids. L-ALA, low-ALA; H-ALA, high-ALA; CON, control

### Blood collection

At the age of 8 weeks, the mice were anaesthetised with isoflurane (Abbott Laboratories). Approximately 100 μl of blood per mouse was collected through the retro-orbital vein by the tubes containing heparin sodium, snap-frozen in liquid nitrogen, and stored at −80 °C until analysis.

### Tissue dissection and processing

The liver and epididymis fat pad were dissected, and the whole mouse and prostate lobes were dissected and weighted as described [16]. Representative whole prostate sections for *Pten*-knockout mice in each group were photographed. All of the prostates and tissues were snap-frozen in liquid nitrogen, and stored at −80 °C until analysis.

### Fatty acid profiles from diets and tissues

Total fatty acids in the diets, blood, liver, epididymis adipose tissue and prostates of mice at the age of 8 weeks were analysed at the same time. Liver, epididymis adipose tissue and prostate samples were each homogenised with 500 μl of methanol before lipid extraction. Total fatty acid extraction was performed as described previously [1], and then 1 μl of solution was analysed on a gas chromatograph (GC2010 plus, Shimadzu, Kyoto, Japan) fitted with a QP2010 ultra mass spectrometer (Shimadzu, Kyoto, Japan) using an Rtx-WAX column (30 m × 0.25 mm i.d. with 0.25 μm thickness) (Restek Corporation, Bellefonte, PA). The temperature programming of the gas chromatography was performed as described previously [17].

### Statistics

All data are presented as means ± SEMs. All analyses were performed using GraphPad Prism 6 (GraphPad Software, Inc) software or SPSS version 23.0 (SPSS Inc., Chicago, IL, USA). Mouse prostate weight data were analysed using a one-way ANOVA model. Prostate weight was adjusted for body weight (mg/25 g). Significant differences between dietary groups were tested using one-way ANOVA for each type of fatty acid analysis. Differences with a *P* value of 0.05 or less were considered significant.

## Results

### An ALA-enriched diet leads to elevated ALA levels in mouse blood and tissues

To assess the efficiency of dietary intake of PUFAs, the percentage of ALA in the blood, liver, epididymal fat pad and prostate of mice fed specified diets were measured and the results are presented in Table 2.

**Table 2:** Percentage of ALA in blood, liver, epididymal fat pad and prostate of *Pten*-knockout mice^1^

	Group^3^, %					
Tissue	CON	L-ALA	H-ALA	EPA	n-3	n-6
blood	ND^2,a^	2.75 ± 0.29^b^	5.98 ± 0.80^c^	ND^a^	ND^a^	ND^a^
liver	ND^a^	3.24 ± 0.52^b^	10.7 ± 1.21^c^	0.22 ± 0.04^a^	0.17 ± 0.04^a^	0.06 ± 0.02^a^
epididymal fat pad	0.12 ± 0.01^a^	9.84 ± 0.29^b^	26.5 ± 0.27^c^	0.59 ± 0.02^a^	0.60 ± 0.05^a^	0.30 ± 0.03^a^
prostate	ND^a^	2.48 ± 0.56^b^	2.72 ± 0.47^b^	ND^a^	ND^a^	0.47 ± 0.07^a^

^1^Values are means ± SEMs (*n* = 6). Labeled means without a common letter differ, *p* < 0.05

^2^ND, not detected

^3^Data expressed as percentage of total fatty acids. L-ALA, low-ALA; H-ALA, high-ALA; CON, control

Our previous study demonstrated that there was a strictly correlated response between increasing EPA concentration in vivo and dietary ALA supplementation in a range up to 5%, but no significant difference in ALA dietary supplementation between 5% and 10% [18]. Therefore, in the present study, all diets contained 13% total fat and the ALA content was 2.5% in the L-ALA diet and 7.5% in the H-ALA diet. The dietary ALA was bioavailable and incorporated into the blood and tissues of the F_2_ mice. The blood lipid ALA levels in mice fed L-ALA and H-ALA diets were 2.75% and 5.98%, respectively. The ALA levels in the liver tissue of mice fed the L-ALA and H-ALA diets were 3.24% and 10.7%, respectively. These levels were higher than those in the blood, perhaps because the liver is a vital organ for lipid metabolism and synthesis of LCPUFAs from ALA [19]. Increasing the level of ALA fed to mice also increased the levels of ALA in the liver and epididymal fat pad. As adipose tissue is important for storing ALA that can be made available to the body when needed [20], the ALA level reached 9.84% in mice fed the L-ALA diet, and 26.5% in those fed the H-ALA diet.

Although the ALA levels increased in a dose-dependent manner in the blood, liver and epididymal fat pad, there were no significant differences in the prostate, with 2.48% for the L-ALA diet and 2.72% for the H-ALA diet. Nevertheless, these results showed that supplementation with ALA increased the levels of ALA in different tissues. Several studies have reported similar findings [21–25], indicating that ALA obtained from the daily diet can be efficiently absorbed and transported by the blood and tissues of mice.

### An ALA-enriched diet is effective in promoting n-3 LCPUFAs levels and lowering AA levels in mouse blood and tissue

ALA is the substrate for the synthesis of LCPUFAs such as EPA, docosapentaenoic acid (DPA; 22:5 n-3) and DHA. Hence, we also examined the effects of different doses of dietary ALA on the concentrations of EPA, DPA and DHA in vivo in the *Pten*-knockout mice (Table 3). The L-ALA and H-ALA diets produced a significant increase in EPA, DPA and DHA levels in the blood, liver and prostate tissues compared with the control and n-6 groups. In the L-ALA group, the level of EPA was 4.05%, 4.90% and 2.04% in the blood, liver and prostate, respectively. The H-ALA diet resulted in an approximately 2-fold increase in its converted product, EPA, in the blood, liver and prostate compared with the L-ALA diet group. However, in the H-ALA diet group, EPA in the blood was found to make up 7.22% of the total fatty acids, only a little less than the EPA and n-3 groups, while the prostate EPA level was close to the two positive control groups, accounting for 3.82% of the total fatty acids. The level of EPA in the liver was 9.68%, which was statistically higher than that in the n-3 group (*p* < 0.001).

**Table 3:** Concentrations of EPA, DPA and DHA in the blood and tissues of *Pten*-knockout mice^1^

		Group^4^, %					
Tissue	Fatty acid	CON	L-ALA	H-ALA	EPA	n-3	n-6
blood	EPA	ND^2,a^	4.05 ± 0.31^b^	7.22 ± 0.28^c^	10.1 ± 0.75^e^	8.90 ± 0.37^d^	ND^a^
	DPA	ND^a^	1.54 ± 0.04^c^	2.14 ± 0.07^d^	2.57 ± 0.18^e^	1.20 ± 0.04^b^	ND^a^
	DHA	1.69 ± 0.11^b^	5.50 ± 0.21^d^	4.49 ± 0.29^c^	6.19 ± 0.24^d^	9.90 ± 0.52^e^	0.78 ± 0.09^a^
	Total^3^	1.69 ± 0.11^a^	11.1 ± 0.46^b^	13.9 ± 0.54^c^	19.8 ± 0.53^d^	20.0 ± 0.83^d^	0.78 ± 0.09^a^
liver	EPA	ND^a^	4.90 ± 0.39^b^	9.68 ± 0.46^c^	8.29 ± 0.83^c^	5.77 ± 0.52^b^	ND^a^
	DPA	0.11 ± 0.00^a^	1.58 ± 0.11^b^	2.18 ± 0.14^c^	3.90 ± 0.45^d^	1.84 ± 0.14^b,c^	0.12 ± 0.02^a^
	DHA	1.70 ± 0.17^a^	10.9 ± 1.24^b^	9.41 ± 0.59^b^	14.0 ± 0.57^c^	22.0 ± 0.77^d^	1.98 ± 0.21^a^
	Total	1.72 ± 0.18^a^	18.8 ± 1.04^b^	21.3 ± 0.80^c^	26.2 ± 1.19^d^	29.6 ± 0.80^e^	2.07 ± 0.22^a^
epididymal fat pad	EPA	ND^a^	0.11 ± 0.01^a^	0.20 ± 0.01^a^	2.31 ± 0.15^c^	1.49 ± 0.18^b^	ND^a^
	DPA	ND^a^	0.17 ± 0.01^a^	0.28 ± 0.01^b^	0.86 ± 0.09^c^	0.93 ± 0.07^c^	ND^a^
	DHA	ND^a^	0.18 ± 0.02^a^	0.16 ± 0.03^a^	0.64 ± 0.07^b^	2.76 ± 0.23^c^	ND^a^
	Total	ND^a^	0.46 ± 0.03^a,b^	0.64 ± 0.03^b^	3.81 ± 0.28^c^	5.17 ± 0.44^d^	ND^a^
prostate	EPA	ND^a^	2.04 ± 0.26^b^	3.82 ± 0.41^c^	4.33 ± 0.96^c^	3.43 ± 0.54^c^	ND^a^
	DPA	ND^a^	2.30 ± 0.28^b^	4.12 ± 0.30^c^	4.14 ± 0.73^c^	1.75 ± 0.13^b^	ND^a^
	DHA	2.18 ± 0.10^a^	4.80 ± 0.54^b^	5.14 ± 0.13^b^	7.58 ± 0.63^c^	9.93 ± 0.72^d^	1.01 ± 0.13^a^
	Total	2.18 ± 0.10^a^	9.15 ± 0.87^b^	13.1 ± 0.28^c^	16.1 ± 2.18^d^	15.1 ± 0.95^c,d^	1.01 ± 0.13^a^

^1^Values are means ± SEMs (*n* = 6). Labeled means without a common letter differ, *p* < 0.05

^2^ND, not detected

^3^Total, the sum of EPA, DPA and DHA

^4^Data expressed as percentage of total fatty acids. L-ALA, low-ALA; H-ALA, high-ALA; CON, control

DPA is an elongated metabolite of EPA and is an intermediary product between EPA and DHA. In both the L-ALA and H-ALA groups, the DPA content was less than the EPA content in the blood and liver; however, the prostate DPA content was similar to the EPA content, which was consistent with the EPA group. In addition, the DPA level in the H-ALA group was higher than that in the L-ALA group in the respective tissues.

Many studies have reported that relatively high consumption of ALA in either animals or humans increases EPA and DPA levels, but not DHA levels, leading to the belief that the major products of ALA metabolism are EPA and DPA [24, 26–28]. In our study, we found that the mice fed ALA had significantly higher levels of DHA in blood and tissues than the control group and n-6 group, although no dose-dependent increase was observed. Among these tissues, the liver had the highest DHA content, with 10.9% in the L-ALA group and 9.41% in the H-ALA group, as much as twice that in the blood and prostate in these groups. In contrast, the epididymal fat tissue contained very small amounts of DHA in the two ALA diets and the n-3 PUFAs concentrations were largely contributed by ALA; the total LCPUFAs detected only accounted for 0.46% and 0.64% in the two ALA groups respectively, far less than the 3.81% in the EPA group and 5.17% in the n-3 group.

**Fig. 1:**
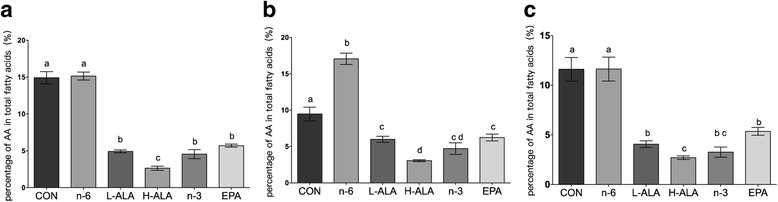
AA levels in blood and tissues of *Pten*-knockout mice. (**a**) blood (**b**) liver (**c**) prostate. Data are means ± SEMs; *n* = 6. Labeled means without a common letter differ, *p* < 0.05. L-ALA, low-ALA; H-ALA, high-ALA; CON, control

Arachidonic acid (AA; 20:4, n-6) is the predominant product of the n-6 pathway and is usually a precursor for the synthesis of eicosanoids, which are involved in pro-inflammatory effects [29]. Therefore, the AA levels in the blood and tissues of *Pten*-knockout mice were analysed (Fig. 1). The highest concentrations were measured in the blood and prostrate of the control and n-6 mice, whereas the n-6 group showed a significant rise in the AA level in the liver compared with the control group (*p* < 0.001). The AA levels in the blood, liver and prostate of the two ALA groups were significantly lower than those of the control and n-6 groups, while the epididymal fat tissue contained only tiny amounts of AA in all groups (data not show). These results suggest that dietary intake of ALA limited the availability of AA in blood and tissues.

### An ALA-enriched diet reduces prostate tumour weight and slows down prostate tumour progression

The levels of total fatty acids measured in tissues indicated that the diets were absorbed equally well in the different groups of *Pten*-knockout mice. To determine whether dietary ALA can modify genetic cancer risk, mice with *Pten* deletion were bred, and fed the specified diets starting from their parental generation. We crossed *Pten* ^*loxp/loxp*^ mice with the ARR2Probasin-*cre* transgenic line, *PB-cre4*, in which the cre recombinase is under the control of a modified mouse prostate-specific probasin promoter [30]. Mice without the tumour suppressor gene *Pten* in the prostate will develop prostate cancer spontaneously, mimicking the disease seen in humans.

**Fig. 2:**
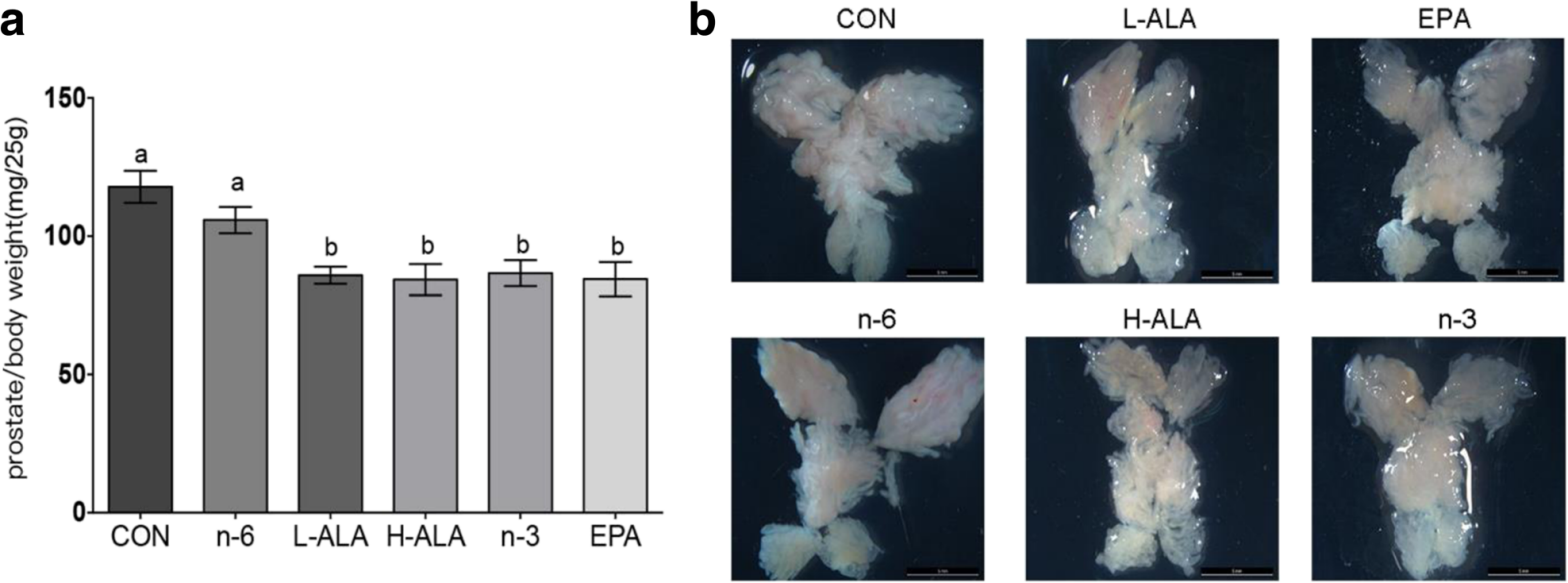
Relative prostate weight and representative gross appearance of the 8-week-old *Pten*-knockout mice. (**a**) Relative prostate weight. Mouse AP, DL and VP lobes were weighed, and the sums were calculated and expressed as mg/25 g body weight. Data are means ± SEMs; *n* ≥ 12. Labeled means without a common letter differ, *p* < 0.05. (**b**) Representative gross appearance. Scale bar: 5 mm. L-ALA, low-ALA; H-ALA, high-ALA; CON, control

We dissected, photographed and weighted the prostate lobes of mice at the age of 2 months, and the relative prostate weight (expressed as mg/25 g body weight) was calculated (Fig. 2). In the wild type groups, the relative prostate weights were in the normal range from 26 to 45 mg/25 g (data not show) regardless of diet, demonstrating that the diet did not affect the prostrate weight of wild type mice. Remarkably, the average prostate weight of *Pten*-knockout mice fed the H-ALA diet was 21.3% lower than that of the control group (*p* < 0.001) and 16.2% lower than that of the n-6 group (*p* = 0.001); and in the L-ALA group, the average weight was 20.1% lower than that of the control group (*p* < 0.001) and 14.9% lower than that of the n-6 group (*p* = 0.002). Both of the ALA dietary groups demonstrated a similar tendency to the EPA and n-3 dietary groups in the modulation of the prostate tumour weight.

## Discussion

As the precusor of n-3 LCPUFAs, the beneficial effects of ALA are still in dispute [2, 9–11]. The primary objective of this investigation was to evaluate the efficiency of the ALA uptake from a vegetable oil source and its subsequent conversion to n-3 LCPUFAs in different mice tissues, and to validate whether it plays a protective role in a prostate cancer animal model.

One of the main metabolic fates of ALA in vivo is its conversion into EPA, DPA and DHA, but studies evaluating efficiency of the conversion of ALA to its longer-chain metabolites have produced highly variable results [31]. Nevertheless, plenty of studies have reached a consensus that increased dietary intake of ALA results in an increase in the levels of ALA, EPA and DPA in plasma or tissues [26, 32, 33], consistent with our observation. In our study, the L-ALA and H-ALA diets contained 17.2% and 42.4% ALA, respectively. The high absolute amount of ALA in the diets may be one explanation for the relatively high rate of transformed EPA in the tissues. Although both LA and ALA use the same enzymes and compete with each other for enzyme availability, ALA is the preferred substrate for delta-6-desaturase, the rate-limiting enzyme in the conversion to EPA [34]. In fact, more than 10 times the amount of LA is required to produce an equal effect on n-3 metabolism to that of ALA on LA elongation [35]. In the present study, the ratio of ALA/LA was found to be 1.3 in the L-ALA diet and 2.5 in the H-ALA diet. It is possible that a high ratio of ALA/LA favours the desaturation and elongation of ALA. Furthermore, the level of EPA was higher in the liver, which may be because the desaturation/elongation pathway in the liver is the most important in terms of supplying ALA metabolites to other tissues [36]. The DHA level in vivo could be influenced by the experimental animal’s age and background diet [37]. Our experimental mice were bred and received the ALA diets for 2 months before they were sacrificed. During the growth stage, a high level of DHA is needed to support membrane turnover and meet the development demands of the cerebral cortex, retina and testis [38–41]. In addition, as the concentration of DHA supplemented in the diet and background diet accounts for the synthesised n-3 LCPUFAs metabolites, a high DHA concentration would be counterproductive [42]. ALA, as the only n-3 PUFA, accounted for 0.60% in the control diet and 0.39% in the n-6 diet, but we detected almost no EPA or DPA, while DHA reached 2.18% and 1.01% in the prostates of the two groups, respectively. Furthermore, there was a significant increase in the DHA levels in the blood and tissues of the two ALA diets compared with the control diet, but no dose-dependent tendency was observed between the L-ALA and H-ALA groups. Taken together with our previous finding that the DHA level in vivo is quite steady, we tend to believe that enhancing the DHA status of mice fed diets containing ALA as the only source of n-3 fatty acids may be efficient, but only when the level of dietary n-3 PUFAs is low or deficient, and DHA tends to remain at a certain level when ALA consumption is above an optimal level [26, 32, 33, 37, 43].

AA is derived from the essential polyunsaturated fatty acid, linoleic acid, which is commonly available in dietary fat. The biosynthesis of eicosanoids depends on the availability of free AA, and contributes to the development and progression of numerous malignant diseases, including prostate cancer. Our data showed that the two ALA groups were in accordance with the n-3 and EPA groups, and AA levels were significantly lower than in the control group in the blood, liver and prostate (Fig. 1a–c). As LA and ALA compete for desaturases and elongases in the lipid synthesis pathway, increased ALA intake leads to reduced AA content and could further lower the biosynthesis of pro-inflammatory eicosanoids including prostaglandins (PGs), such as PGE_2_; leukotrienes (LTs); and thromboxanes. The effect and signal transduction of AA and its lipid mediators on the organism have been well characterized, including cancer [44]. For example, PGE_2_ was already reported to promote tumor growth through induced *c-fos* expression [45], downregulating these prostanoids might provide a new avenue of investigation for the inhibition of cancer. Thus, as an AA antagonist, ALA is more biologically potent than that from fish oil.

The average prostate weight in the L-ALA and H-ALA groups (86.6 and 85.2 mg/25 g, respectively) was lower than that in the control and n-6 groups (108 and 102 mg/25 g, respectively), and similar to that in the EPA and n-3 groups (82.3 and 88.5 mg/25 g, respectively). These findings demonstrate that the diets rich in ALA supplementation decreased the prostate tumour weight in *Pten*-knockout mice, with an effect similar to that of the EPA and n-3 supplementation groups. A recent study that explored non-tumour-bearing and tumour-bearing Wistar rats supplemented with fish oil or Oro Inca® oil (rich in ALA) also showed that oil rich in ALA had a similar immune modulation effect to that of fish oil [46].

The results of our prostate fatty acid analysis showed that the levels of both EPA and total LCPUFAs in the H-ALA group were not significantly different from those in the EPA group (*p* = 0.50 for EPA, *p* = 0.08 for LCPUFAs) or the n-3 group (*p* = 0.62 for EPA, *p* = 0.24 for LCPUFAs). The effect of ALA on prostate cancer progression may be partly explained by the fact that n-3 LCPUFAs converted in vivo can maintain adequate concentrations in cell membranes, and thus optimal tissue function, because an animal study found that dietary n-3 LCPUFAs reduced prostate tumour growth, slowed histopathological progression and increased survival [1]. We also found that the L-ALA group demonstrated a similar effect in modulating the relative prostate weight to that of the H-ALA group and the two positive control groups. However, in the prostate tissue, the amount of EPA converted from ALA in the L-ALA group was only half that in the H-ALA group and the two positive control groups, and the total LCPUFAs also showed a significant difference compared with these three groups. As the lowest effective dose of EPA and DHA for cancer prevention is unclear [47], it is possible that the concentration of n-3 LCPUFAs in vivo in the L-ALA diet had reached the threshold for action and/or ALA directly interacted with nuclear receptors and transcription factors, which play an important role in the regulation of lipid homeostasis. However, an inverse association was previously observed between the ALA level of adipose breast tissue and breast cancer with biopsies of adipose breast tissue obtained from 123 women, but no association was found between saturates, monounsaturates, n-6 LCPUFAs, n-3 LCPUFAs and the disease [48]. In addition, our data showed a lower AA content in blood, liver and prostate of the mice in the two ALA groups. In vivo and in vitro experiments have demonstrated that the AA metabolites, particularly those generated through the LOX, such as 12-HETE, are critical to prostate cancer progression [49]. PGE_2_ is proved to result in increased proliferation and decreased apoptosis in androgen sensitive and insensitive prostate cancer cells, including LNCaP and PC-3 [50]. Thus, a lower AA content in vivo could result in less eicosanoids, which may contribute to the protective effect of ALA in prostate cancer. It will be interesting to explore the possible mechanism of action of ALA’s protective effect on prostate cancer, and whether it has an independent function or needs to be converted into LCPUFAs to exert its stimulatory effect.

Our animal experiment showed an equivalent effect of certain doses of ALA and marine-derived oil on prostate cancer. However, mice fed ALA diets exhibited significantly higher levels of plasma total cholesterol and LDL-cholesterol than the n-3 and EPA groups. In addition, the H-ALA diet produced a significant increase in serum alanine aminotransferase compared with controls.

## Conclusion

Plant-derived ALA has received increasing interest because it is available from extensive sources and is transformed into n-3 LCPUFAs, and thus may have metabolic, functional and health benefits. We found that dietary ALA triggered significant increases in ALA, EPA, DPA and DHA levels and significant decreases in AA levels in blood, liver and prostate tissues compared with the control group when the mice were in the growth stage. Moreover, a dose-dependent effect was observed for ALA and EPA and DPA, but not DHA. To our knowledge, this study is the first to demonstrate that dietary ALA can delay prostate tumour formation and progression as the marine-derived oil. ALA, a gift from the land, has been proven to be precious for its potential beneficial effect on prostate cancer.

## Additional file

Additional file 1: Table S1. Composition of the experimental diets. (DOCX 17 kb)

## Abbreviations

AA: arachidonic acid
ALA: α-linolenic Acid
AP: anterior prostate
CON: control
DHA: docosahexaenoic acid
DL: dorsal and lateral prostate
DPA: docosapentaenoic acid
EPA: eicosapentaenoic acid
H-ALA diet: high-ALA diet
LA: linoleic acid
L-ALA diet: low-ALA diet
LCPUFA: long-chain polyunsaturated fatty acid
PGE_2_: prostaglandin E_2_
VP: ventral prostate
