# Supplementary material for: Dietary supplementation of α-linolenic acid induced conversion of n-3 LCPUFAs and reduced prostate cancer growth in a mouse model
Source: Lipids Health Dis. 2017 Jul 11;16:136. doi: 10.1186/s12944-017-0529-z (PMC5505143; doi:10.1186/s12944-017-0529-z)
Supplement: Additional file 1: Table S1. — Composition of the experimental diets. (DOCX 17 kb) [file 12944_2017_529_MOESM1_ESM.docx]

**Additional file 1**

**Table S1 Composition of the experimental diets**

| Diets | CON | L-ALA | H-ALA | EPA | n-3 | n-6 |
| --- | --- | --- | --- | --- | --- | --- |
| ingredient | g/100g | g/100g | g/100g | g/100g | g/100g | g/100g |
| Palm oil | 6.50 | 7.82 | 3.09 | 7.06 | 7.58 | 6.50 |
| Safflower oil | 0 | 0 | 0 | 0.47 | 0 | 6.50 |
| Olive oil | 6.50 | 2.16 | 0.85 | 2.83 | 2.84 | 0 |
| Flaxseed oil | 0 | 3.02 | 9.06 | 0 | 0 | 0 |
| DHA oil | 0 | 0 | 0 | 0 | 1.26 | 0 |
| EPA oil | 0 | 0 | 0 | 2.64 | 1.32 | 0 |
| Wheat flour | 30 | | | | | |
| Dextrin | 10  10  10  10  10  10 | | | | | |
| Sucrose | 10  10  10  10  10  10 | | | | | |
| Casein | 10  10  10  10  10  10 | | | | | |
| Lactalbumin | 10  10  10  10  10  10 | | | | | |
| Alphacel | 11  11  11  11  11  11 | | | | | |
| β-sitosterol | 0.015  0.015  0.015  0.015  0.015  0.015 | | | | | |
| Mineral mix | 4.7  4.7  4.7  4.7  4.7  4.7 | | | | | |
| Vitamin mix | 1  1  1  1  1  1 | | | | | |
| Choline bitartrate | 0.25  0.25  0.25  0.25  0.25  0.25 | | | | | |
| Tenox 20A | 0.006  0.006  0.006  0.006  0.006  0.006 | | | | | |
| % Fat | 13 | | | | | |
| % Protein | 24.5 | | | | | |
| % Carbohydrate | 46.5 | | | | | |
| Kcal/100g | 397 | | | | | |

Adapted with permission from [1].

L-ALA, low-ALA; H-ALA, high-ALA; CON, control.
